# Performance Analysis of Single-Frequency MPPSK Integrated System for Ranging Applications

**DOI:** 10.1155/2014/670346

**Published:** 2014-07-21

**Authors:** Yu Yao, Lenan Wu

**Affiliations:** School of Information Science and Engineering, Southeast University, Nanjing 210096, China

## Abstract

The dual-frequency MPPSK-MODEM is a flexible platform. When ranging accuracy request is low or platform is particularly limited by power, the platform would perform both data transmission and range measurement with single-frequency modes. In this paper, the ranging resolution of MPPSK pulse waveforms with the match filter and impacting filter processing are discussed, respectively. Also, the parameter selection of MPPSK modulator for ranging is considered. In particular, requirements that allow for employing such special parameter values for range measurements with high accuracy and high range are investigated. Moreover, high repetition frequency (HRF) biphase code MPPSK pulse train base on *m* sequence is presented, and the ranging accuracy of the proposed signal with the match filter processing is deduced. In addition to theoretical considerations, the paper presents system simulations and measurement results of single-frequency MPPSK integrated systems, demonstrating the high-performance of ranging applications.

## 1. Introduction

To achieve very high spectrum efficiency without excessively penalizing the power efficiency, the efficient modulation technique named M-ary position phase shift keying (MPPSK) was presented [1, 2]. Comparing to EBPSK, proposed by Wu et al. [3, 4], the MPPSK utilizes M-ary information symbols to directly control the positions of phase transition of sinusoidal carrier in each symbol cycle. These modulation techniques offer a number of advantages, such as ultranarrow bandwidth, very high transmission efficiency, and high and adjustable data rate [5, 6].

Then a dual-frequency MPPSK-MODEM integrated platform is creatively proposed to overcome the ranging ambiguity of dual-frequency continuous wave (CW) radar [7, 8]. The platform can be also used for the digital video broadcasting satellite services [9]. Meanwhile, the receiver of the system is equipped with a much narrower pass-band filter named digital impacting filter (DIF), it can extract the tiny phase modulation to ease demodulation. Literatures [10–12] had mentioned the special principle of DIF elaborately. Such a system will provide two functions on a single hardware platform of MPPSK modems, which is a flexible one. When ranging accuracy request is low or platform is particularly affected by power limitations, pulse train MPPSK modulator 1 will stop transmitting CW in carrier frequency *f*_0_, and receiver 1, which is mainly composed of phase discriminator (PD), also stops working. The platform would perform both data transmission and range measurement with single-frequency modem [13]. In this paper, the ranging resolution of the single-frequency MPPSK pulse waveforms with the match filter and impacting filter processing are discussed, respectively. High repetition frequency (HRF) biphase code MPPSK pulse train base on *m* sequence is presented, and the ranging accuracy of the proposed signal with the match filter processing is deduced.

The rest of this paper is organized as follows.  Section 2 introduces MPPSK pulse waveforms, including the waveforms with special modulation parameter values *M* = 3 and *K* = *N*/2.  Section 3 analyses the ranging accuracy of single MPPSK pulse waveform with the impacting filter processing. In Section 4, HRF biphase code MPPSK pulse train base on *m* sequence is presented; also, the ranging resolution of the proposed signal with the match filter processing is deduced. Some indicative simulation results and performance analysis are presented in Section 5. And finally, Section 6 gives the conclusion of the paper.

## 2. MPPSK Pulse Waveforms

The modulated MPPSK pulse waveforms are defined by [1], and the simplified expression is presented in the paper as follows:

(1) $$\begin{array}{c} s_{1}\left(t\right)=\{\begin{array}{cc} \sin\left(2\pi f_{1}t\right),\;0\leq t<NT_{1},\;k=0, & \\ \{\begin{array}{cc} \sin\left(2\pi f_{1}t\right), & 0\leq t\leq \left(k-1\right)KT_{1},\\ -\sin\left(2\pi f_{1}t\right), & \left(k-1\right)KT_{1}<t<kKT_{1},\\ \sin\left(2\pi f_{1}t\right), & kKT_{1}\leq t<NT_{1}, \end{array} & \\ 1\leq k\leq M-1, & \end{array} \end{array}$$

where *f*_1_ and *T*_1_ represent the carrier frequency and the carrier period, respectively. *K* and *N* stand for the number of the carrier period in each time slot and the number of the carrier period in each symbol, respectively. *m*  (*m* = 0,1,…, *M* − 1) is M-ary (*M* ≥ 2) source symbol.

Modulation parameters set special values *M* = 3 and *K* = *N*/2; MPPSK pulse waveforms corresponding to nonzero symbols, that is, “1” to “2”, are subdivided into a number of subpulses each of duration *KT*_1_, where *NT*_1_ is the pulse width and 2 is the number of subpulses. A MPPSK-MODEM integrated system transmits a MPPSK pulse with width *NT*_1_, which is equivalent to be coded using phase modulation with Barker code of length 2. And the simplified expression corresponding to nonzero symbols (“1” and “2”) is presented in the paper as follows:

(2) ui(t)={sin(2πf1t),0≤t≤(k−1)KT1,−sin(2πf1t),(k−1)KT1<t<kKT1,sin(2πf1t),kKT1≤t<NT1,1≤k≤2.

**Figure 1:**
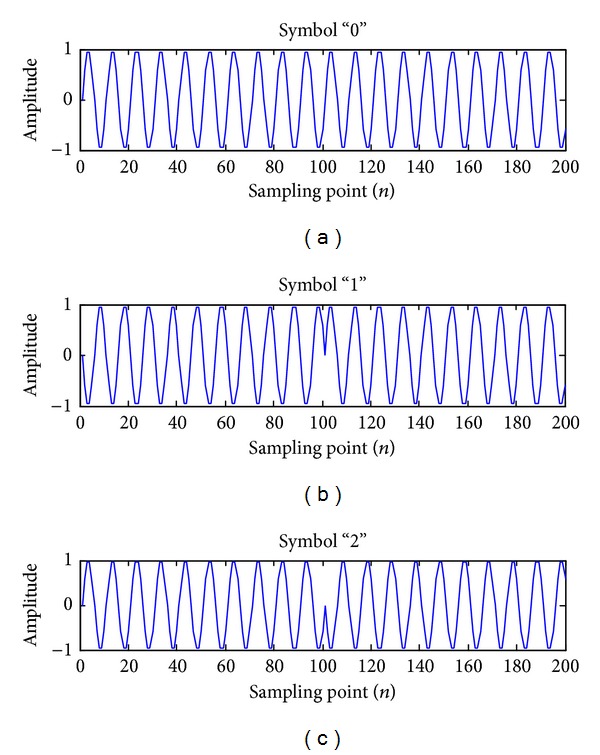
3PPSK modulated waveforms.

The waveforms of 3PPSK modulation are illustrated as shown in Figure 1. The coefficient for the *x*-axis is the index of a certain sample point.

The modulation waveform for symbol “0” is sinusoidal as shown in Figure 1(a); Figure 1(b) illustrates the modulation waveform for symbol “1” with the phase hopping during the first ten carrier periods (from 0 to 100), and the next (from 100 to 200) is for symbol “2” in Figure 1(c).

3PPSK pulse waveforms corresponding to symbols “1” and “2” are in-pulse phase coded waveforms. The time-bandwidth product and pulse compression ratio are equal to the number of subpulses in the waveform. The number of elements in the code is 2. The range resolution is proportional to the time duration of one subpulse *KT*_1_, which is much higher compared to that of an uncoded pulse waveform corresponding to symbol “ 0” with the same duration *NT*_1_.

The dual-frequency MPPSK-MODEM is a flexible platform [7]. When ranging accuracy request is low or platform is particularly affected by power limitations, pulse train MPPSK modulator 1 would stop transmitting CW in carrier frequency *f*_0_, and pulse train MPPSK modulator 2 transmits MPPSK signals in carrier frequency *f*_1_ alone, which is efficient regarding the amount of information they convey on the bandwidth they occupied. At the receiver, the MPPSK signals in carrier frequency *f*_1_ are demodulated by VBR-MPPSK demodulator alone.

## 3. Impacting Filter Demodulation

The impacting filter (IF) is a special digital infinite impulse response (IIR) filter, with the feature of “notch-frequency selection” in an extremely narrow pass-band [11]. It highlights the difference of the modulation waveform. In the following real simulation, we assume the impacting filter formed by one pair of conjugate zeros and four pairs of adjacent conjugate poles. The expression and related parameter of the IF are given in [12]:

(3) $$\begin{array}{c} H\left(z\right)=\frac{1+b_{1}\cdot z^{-1}+z^{-2}}{1-\sum_{i\;=\;1}^{2n}a_{i}\cdot z^{-i}}, \end{array}$$

where *n* is the pair number of the conjugate poles. In order to demodulate MPPSK signals, the zero parameters of the IF are selected as *b*_1_ = −1.6181733185991785, and the pole parameters of the IF in this paper are selected as

(4) a1=−6.1150669443734404,  a2=17.593270854070781,a3=−30.66190141963812,    a4=35.258220132970798,a5=−27.343924194038685,  a6=13.991777506187015,a7=−4.3370740838799371,  a8=0.63250878596652416.

**Figure 2:**
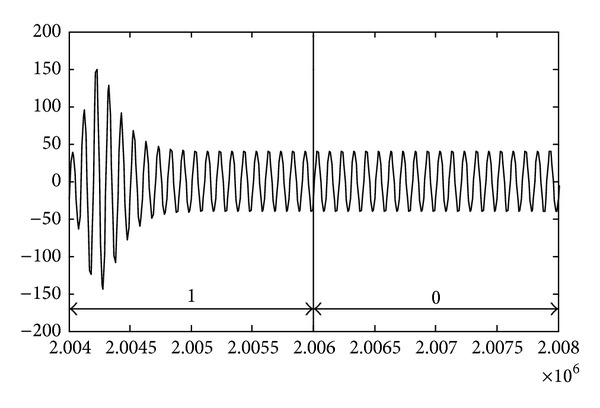
The waveform characteristics of amplitude impacting.

The proposed filter has a very narrow bandwidth, and the IF would retain the signal characteristics and reduce the noise. When MMPSK modulated signals pass the impacting filter, the mechanism of IF is to transform the tiny waveform difference into amplitude impacting. After passing IF, the MPPSK pulse echo signals would produce a significant amplitude impacting within the time duration of phase modulation *KT*_1_. The waveform characteristics of the output of IF are shown in Figure 2.

From Figure 2, for MPPSK pulse waveform corresponding to nonzero symbol with *K* = 3, the phase hopping can be converted into amplitude impacting within phase modulation duration *KT*_1_. After envelope detecting and threshold decision, the width of amplitude impacting is approximately equal to *KT*_1_. According to the impacting filter processing, it can be concluded that the ranging accuracy of single-frequency MPPSK-MODEM integrated system is proportional to the time duration of phase modulation.

So primary advantages of the proposed MPPSK-MODEM are summarized as follows.

- MPPSK is directly RF modulated and their rate limit will be the single cycle modulation of its carrier, which implies bit rates could be as high as the RF is. At the same time, it only requires sidebands much lower than other modulations and frequency hop to vacant spectrum.
- For a given RF carrier of *f*_1_, the shorter the interval *K* is, the higher the range resolution is. On the contrary, it is easy to believe that the longer the *T* is, the higher the energy will be. Therefore, MPPSK modulation parameter *K* may have the potential to adapt with ranging precision.
- The carrier synchronous nature of MPPSK also bests other broad bandwidth modulations where carrier has been suppressed, leading to an easier detection and a simpler implementation. While prior schemes tried to move as much power as possible into the sidebands and away from the carrier signal, MPPSK does the opposite, placing most of the power in the carrier to keep sideband energy emissions negligible.

## 4. Match Filter Demodulation

**Figure 3:**
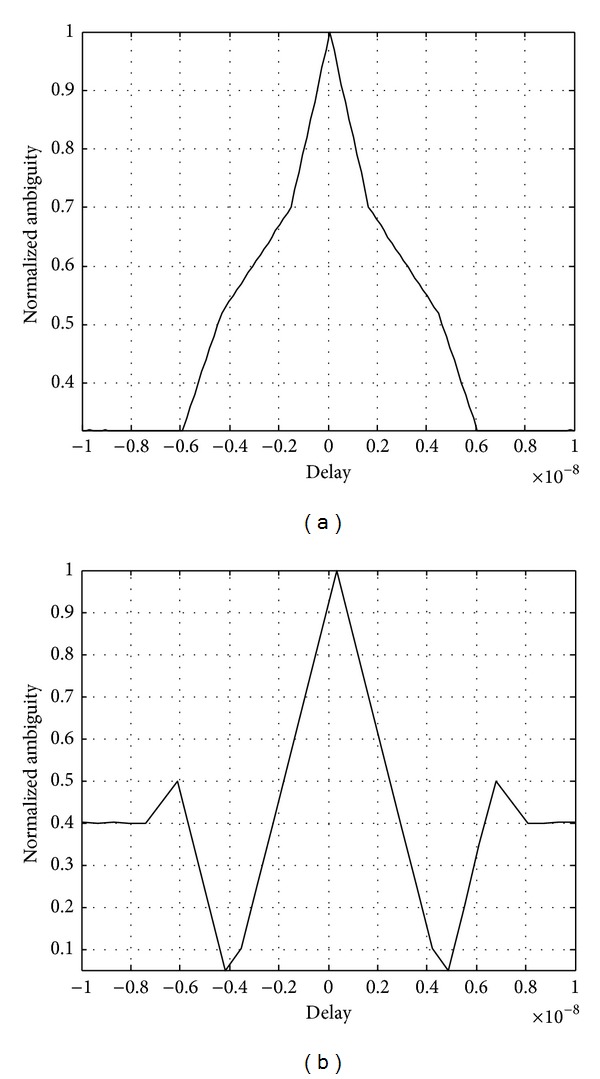
Range ambiguity function of MPPSK pulse.

We consider the ranging accuracy of single-frequency MPPSK integrated system with the match filter processing method. In fact, efficient modulation signal corresponding to nonzero symbol is intrapulse phase code compression signal. Taking single MPPSK pulse waveform corresponding to symbol “1” as an example, it is subdivided into 2 subpulses. The width of MPPSK pulse is *NT*_1_. Time durations of the first and the second subpulse are *KT*_1_ and (*N* − *K*)*T*_1_, respectively. And in general, set the parameters as follows: carrier frequency is *f*_1_ = 10 GHz; modulation parameters are *K* = 5 and *N* = 100. The range ambiguity function of single MPPSK pulse corresponding to symbol “1” with *K* = 5 is illustrated as shown in Figure 3.

As shown in Figure 3(a), for symbol “1,” when these parameters set general values *K* ≠ *N*/2, the number of subpulses is 2. The width of the main peak is equal to about *NT*_1_. So the ranging resolution of single MPPSK waveform is approximately proportional to pulse duration *NT*_1_.

And a single-frequency MPPSK integrated system transmits a MPPSK pulse signal with width *NT*_1_. The parameters assume special values *K* = *N*/2. The range ambiguity function of the MPPSK pulse signal corresponding to symbol “1” with *K* = *N*/2 is shown in Figure 3(b). According to the match filter processing, when the parameters set special values *M* = 3 and *K* = *N*/2, the width of the main peak is equal to about *KT*_1_, and the ranging resolution of single MPPSK waveform is proportional to time duration of phase modulation *KT*_1_. The ranging accuracy of single MPPSK pulse is highest.

High repetition frequency (HRF) coherent pulse train signal is frequently used in the field of high-performance radar [14]. The waveform has the capability of high range resolution the same as narrow pulse, and it also has the advantages of fine frequency resolution because of quite long durations. Transmit-receive community antenna system could easily be achieved with pulse train signals. It does not have the problem of power leakage in the transmitter. Complex envelop of HRF coherent pulse train can be expressed as

(5) $$\begin{array}{c} s\left(t\right)=\frac{1}{\sqrt{N_{p}}}\overset{N_{p}-1}{\underset{i\;=\;0}{\sum }}u_{1}\left(t-iT_{r}\right), \end{array}$$

where *N*_*p*_ is the number of pulses, *T*_*r*_ is pulse interval, and the ambiguity function of a HRF coherent pulse train signal is

(6) |χ(τ,fd)|=1Np∑q = −(Np−1)Np−1|χ1(τ−qTr,fd)|·|sin[πfd(Np−|q|Tr)]sin(πfdTr)|.

Range ambiguity function is

(7) $$\begin{array}{c} \left|\chi \left(\tau ,0\right)\right|=\left|\overset{N_{p}-1}{\underset{q\;=\;-(N_{p}-1)}{\sum }}\left(1-\frac{\left|q\right|}{N_{p}}\right)\left(1-\frac{\left|\tau -qT_{r}\right|}{t_{p}}\right)\right|. \end{array}$$

Velocity ambiguity function is

(8) $$\begin{array}{c} \left|\chi \left(0,f_{d}\right)\right|=\left|\frac{1}{N_{p}}\frac{\sin\left(\pi f_{d}t_{p}\right)}{\pi f_{d}t_{p}}\cdot \frac{\sin\left(\pi f_{d}N_{p}T_{r}\right)}{\sin\left(\pi f_{d}T_{r}\right)}\right|, \end{array}$$

where *t*_*p*_ is the width of single pulse. Set the parameters as follows: pulse interval is *T*_*r*_ = 10*t*_*p*_, the number of pulses is *N*_*p*_ = 5, and the range and velocity ambiguity function of coherent pulse train signal are shown in Figure 5.

**Figure 4:**
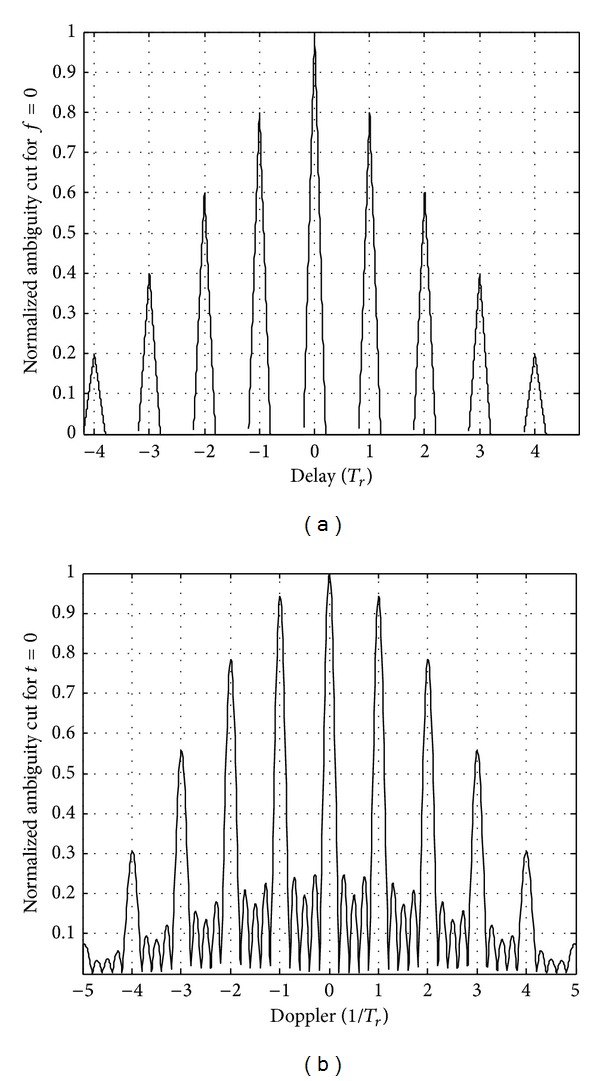
The range and velocity ambiguity function of coherent pulse train.

As shown in Figure 4(a), for ranging ambiguity function, the sidelobe interval is *T*_*r*_, the average sidelobe height is 1/*N*_*p*_, the relative height of any sidelobes is |*N*_*p*_ − |*n*|*N*_*p*_|, and (−(*N*_*p*_ − 1) ≤ *n* ≤ *N*_*p*_ − 1). It has low ranging measurement resolution and low measurement range. And for velocity ambiguity function, the sidelobe interval is 1/*T*_*r*_ as shown in Figure 4(b). So the performance of HRF pulse train signal is unsatisfactory.

**Figure 5:**
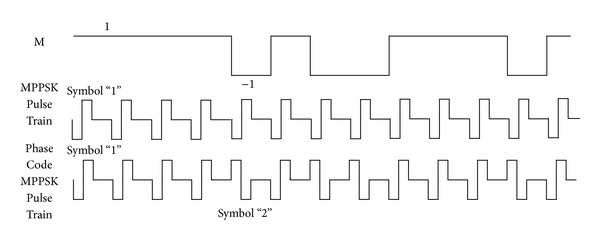
HRF biphase code MPPSK pulse train.

A compression ranging system transmits biphase code CW signal [15, 16], which is coded using phase modulation with *m* sequence. The ranging performance is getting better with the increase of the number of pulses *N*_*p*_. Therefore, combining the merits of biphase code CW signal base on *m* sequence and coherent pulse train, a new kind of signal, named HRF biphase codes MPPSK pulse train base on *m* sequence, is proposed in this paper. It is possible to expand the measuring range and to improve the accuracy of measurement. The proposed signal is shown in Figure 5.

**Figure 6:**
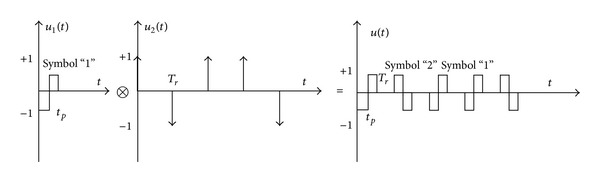
Interpulse biphase code signal.

The waveforms with phase jump information corresponding to nonzero symbols are only considered in this paper. HRF biphase code MPPSK pulse train is a kind of intrapulse and interpulse compression signal. The expression of the proposed signal can be expressed as

(9) $$\begin{array}{c} s\left(t\right)=\frac{1}{\sqrt{N_{p}}}\overset{+\infty }{\underset{i\;=\;0}{\sum }}c_{k}u_{1}\left(t-iT_{r}\right), \end{array}$$

where *c*_*k*_ = *a*_*k*_ *e* ^*jθ*_*k*_^, *u*_1_(*t*) is the amplitude envelope of MPPSK pulse waveform corresponding to symbol “1,” which is in-pulse compression signals. *T*_*r*_ is pulse interval. *N*_*p*_ is the number of pulses in a period of *m* sequence. $1/\sqrt{N_{p}}$ is constant factor for energy normalization operation. *t*_*p*_ = *NT*_1_ is the width of single MPPSK pulse. *t*_*p*_′ = *KT*_1_ is the equivalent width of single MPPSK pulse. The convolution process is shown in Figure 6.

According to the definition of ambiguity function, the complex envelop of the proposed signal can be expressed as

(10) $$\begin{array}{c} u\left(t\right)=u_{1}\left(t\right)⊗u_{2}\left(t\right), \end{array}$$

where *u*_1_(*t*) is the equivalent complex envelop of single MPPSK pulse:

(11) u1(t)={1NT1/2,0<t<NT12,0,others,u2(t)={∑k = 0Np−1ckδ(t−kTr),0≤t≤NpTru3(t−NpTr),others,u3=∑k = 0Np−1ckδ(t−kTr), 0≤t≤NpTr,χ(τ,fd)=χ1(τ,fd)⊗χ2(τ,fd)=∑m = −(−Np−1)Np−1χ1(τ−mTr,fd)χ2(mTr,fd),

where *τ* is time delay and *f*_*d*_ is Doppler frequency shift. *χ*_1_(*τ*, *f*_*d*_) and *χ*_2_(*τ*, *f*_*d*_) are ambiguity functions of *u*_1_(*t*) and *u*_2_(*t*), separately. Consider

(12) χ1(τ,fd)  ={sinπfd(NT1/2−|τ|)πfd(NT1/2−|τ|)(1−2|τ|NT1),|τ|≤NT120,others,χ2(mTr,fd)=∑k = 0Np−1ckck+|m|ej2πfdkTr, |m|≤Np−1.

And ranging ambiguity function is

(13) $$\begin{array}{c} \chi_{u_{K}}\left(\tau ,0\right)=\overset{N_{P}-1}{\underset{m\;=\;-\left(N_{P}-1\right)}{\sum }}\left(1-\frac{\left|\tau -mT_{r}\right|}{T_{r}}\right)\chi_{2}\left(mT_{r},0\right). \end{array}$$

## 5. Simulation

### 5.1. Impacting Filter Processing Method

In this section, the ranging performance of single-frequency MPPSK integrated system is simulated. Firstly, we consider the ranging accuracy of MPPSK pulse signals with the impacting filter processing. In Table 1, a summary of the important parameters of the simulation model is provided.

**Figure 7:**
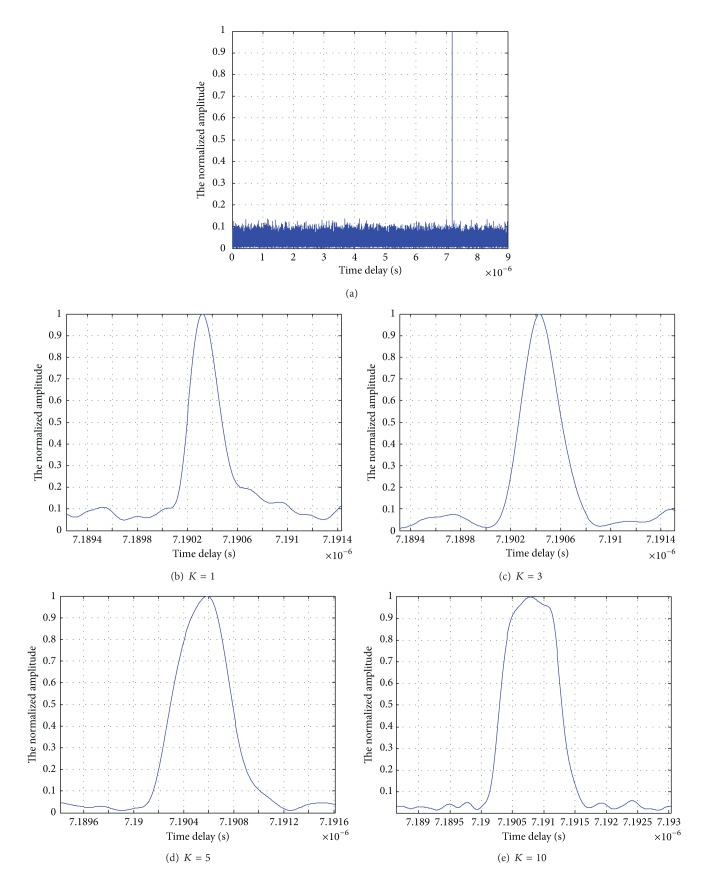
Amplitude impacting of echo signal and the local enlarging graph for *K* = 1,3, 5,10.

The amplitude impacting of echo signal is shown in Figure 7(a), and Figures 7(b), 7(c), 7(d), and 7(e) are the local enlarging graph for modulation parameters *K* = 1,3, 5,10. The simulation experiment is in the AWGN channels (SNR = 2 dB).

The widths of impacting envelope with modulation parameters *K* = 1, 3, 5, 10 are about 2.3 × 10^−10^ s, 4.3 × 10^−10^ s, 6 × 10^−10^ s, and 13 × 10^−10^ s as shown in Figures 7(b), 7(c), 7(d), and 7(e), respectively. Thus, it is concluded that the width of impacting envelope is approximately equal to *KT*_1_ and will be getting greater increasing the value of *K*.

**Figure 8:**
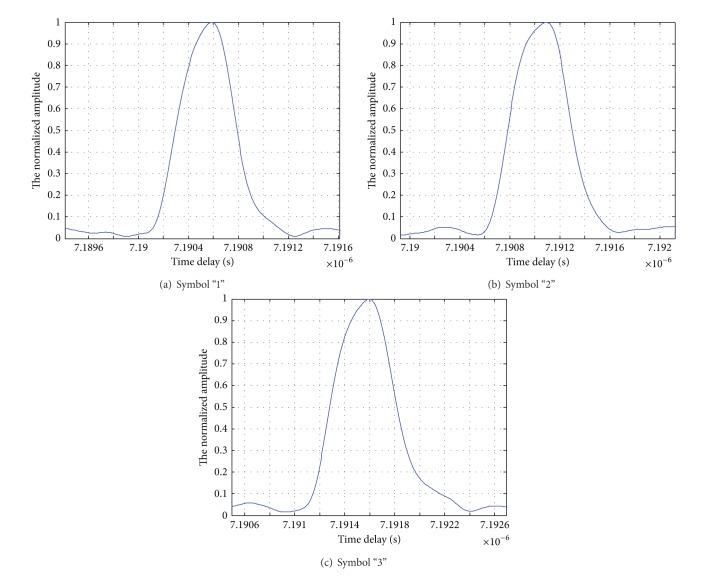
Local enlarging graph for symbols “1,” “2,” and “3.”

The amplitude impacting of MPPSK pulse echo signals corresponding to symbols “1,” “2,” and “3” with *K* = 5 is shown in Figure 8.

As shown in Figure 8, the time delay of amplitude impacting corresponding to symbol “1” is from 7.1903 × 10^−6^ s to 7.1909 × 10^−6^ s, symbol “2” is from 7.1908 × 10^−6^ s to 7.1914 × 10^−6^ s, and symbol “3” is from 7.1912 × 10^−6^ s to 7.1918 × 10^−6^ s. For different nonzero symbols, the time delays of amplitude impacting are different too, but the widths of impacting envelop are the same. Therefore, according to the impacting filter processing method, it can be concluded that the ranging resolution of the single-frequency MPPSK-MODEM integrated system is hence proportional to the time duration of phase modulation corresponding to symbol “M”; the ranging accuracy becomes higher with the decrease of the value of *K*.

However, it is important to choose a relatively larger value of *K*, in order to ensure that the amplitude impacting of echo signals can be detected in the low SNR. In Table 2, a summary of the important parameters of the simulation model is provided.

**Figure 9:**
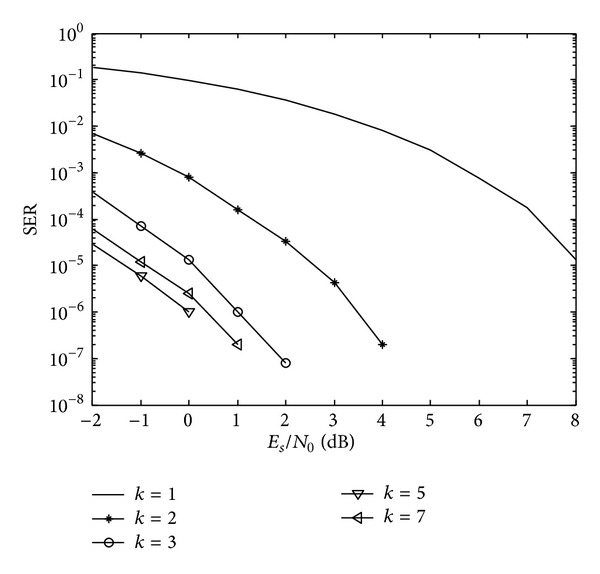
Comparative SER results of *K* = 1, 2, 3, 5, 7.

Figure 9 shows a plot of SER as a function of SNR for modulation parameters *K* = 1, 2, 3, 5, 7.

As shown in Figure 9, it illustrates the SER in different value of *K*; the simulation shows that, at SER = 10^−5^, the SNR performance of single-frequency MPPSK-MODEM system with *K* = 5 may be improved by approximately 9 dB, 4 dB, and 1.2 dB as compared with *K* = 1,2, 3, respectively. The result of the integrated system with *K* = 5 is slightly better than parameters *K* = 7 in order to obtain the same SER performance. So it is important to trade off the parameters selection of MPPSK.

### 5.2. Match Filter Processing Method

**Figure 10:**
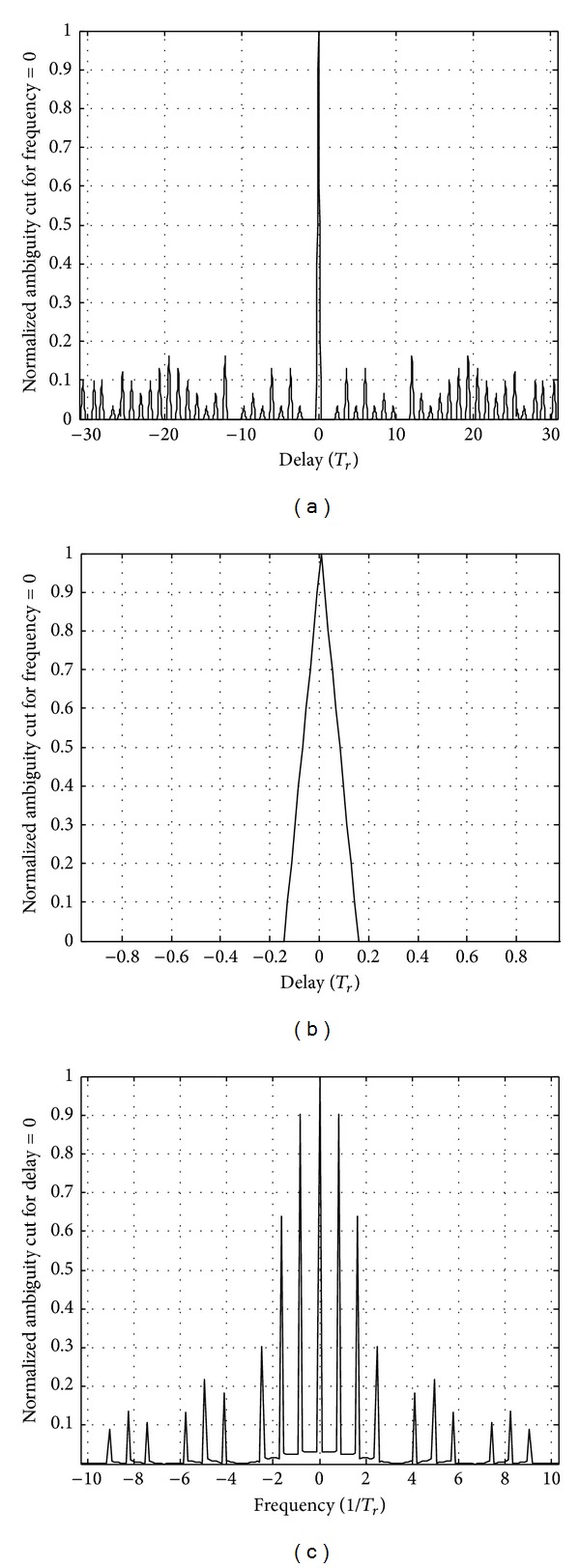
The ranging and velocity ambiguity function for *N*_*p*_ = 31 and *T*_*r*_ = 4*t*_*p*_.

Next, we consider the ranging and velocity resolution of HRF biphase code MPPSK pulse train base on *m* sequence with the match filter processing method. Set the parameters as follows: the number of pulses is *N*_*p*_ = 31, the pulse interval is *T*_*r*_ = 4*t*_*p*_, and the ranging and velocity ambiguity function of the proposed signal are shown in Figure 10.

From Figure 10(a), the characteristics of ranging ambiguity function of the proposed signal are similar to that of biphase code CW signal base on *m* sequence. Intervals between wave peaks are *N*_*p*_ *T*_*r*_. The optimal ratio of major to minor lobes is approximately equal to the number of pulses in a period of *m* sequence. But the energy in the sidelobe region is slight uneven distribution. Main peak is very steep, and the width of main peak is *t*_*p*_′ = *KT*_1_ as shown in Figure 10(b).

As shown in Figure 10(c), the characteristics of velocity ambiguity function of the signal are similar to that of HRF coherent pulse train. Its envelop is Sinc function, the width of main lobes is 1/*N*_*p*_ *T*_*r*_, and intervals between wave peaks are 1/*T*_*r*_. There are higher sidelobe levels on the frequency axis. The proposed signal is Doppler sensitive signal.

**Figure 11:**
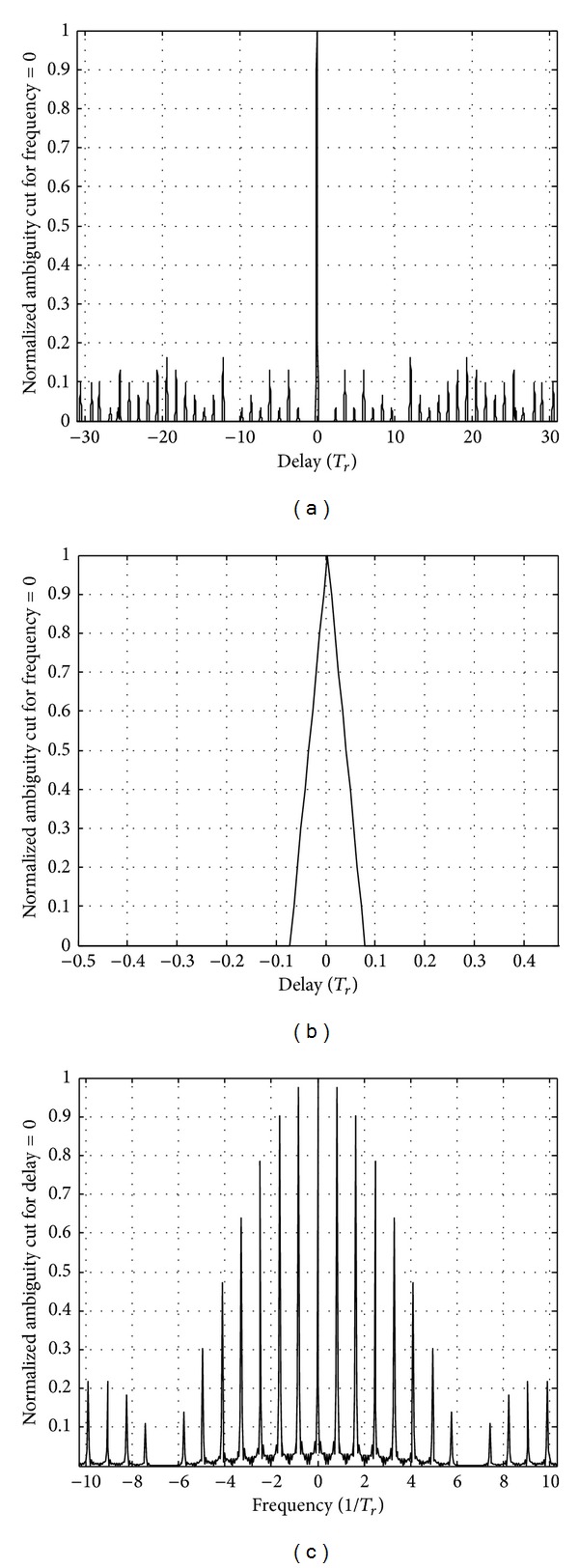
The ranging and velocity ambiguity function for *N*_*p*_ = 31 and *T*_*r*_ = 7*t*_*p*_.

Set the parameters as follows: the number of pulses is *N*_*p*_ = 31 and the pulse interval is *T*_*r*_ = 7*t*_*p*_. And the ranging and velocity ambiguity function of the proposed signal are shown in Figure 11.

Because of the same number of pulses, for ranging ambiguity function, both intervals between wave peaks are same as shown in Figures 10(b) and 11(b). Sidelobe levels of velocity ambiguity function with *T*_*r*_ = 7*t*_*p*_ are higher than *T*_*r*_ = 4*t*_*p*_ as shown in Figures 10(c) and 11(c).

**Figure 12:**
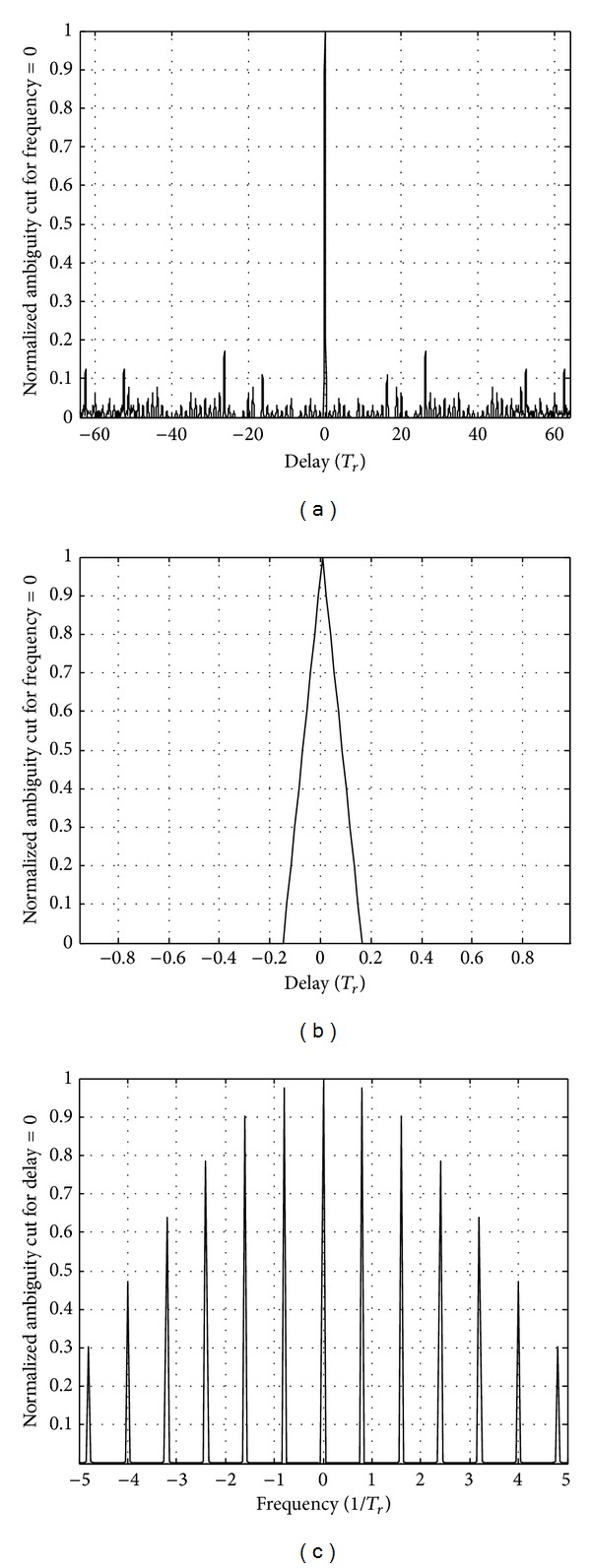
The ranging and velocity ambiguity function for *N*_*p*_ = 64 and *T*_*r*_ = 4*t*_*p*_.

Set the parameters as follows: the number of pulses is *N*_*p*_ = 64, the pulse interval is *T*_*r*_ = 4*t*_*p*_, and the ranging and velocity ambiguity function of the proposed signal are shown in Figure 12.

From Figure 12(a), for ranging ambiguity function, intervals between wave peaks with the number of pulses *N*_*p*_ = 64 are larger than *N*_*p*_ = 32; both widths of main peak are same as shown in Figures 11(b) and 12(b). For velocity ambiguity function, the width of main lobes with *N*_*p*_ = 64 is steeper than *N*_*p*_ = 32 as shown in Figures 11(b) and 12(b).

The ranging performance is getting better with the increase of the number of pulses *N*_*p*_ and pulse interval *T*_*r*_. It means that the measurement range of the proposed signal is much larger than coherent pulse train for same PRF. The main peak width of the proposed signal is narrower than coherent pulse train for same pulse width. So the ranging accuracy of match filter processing method is higher than previously proposed schemes.

The proposed signal has advantages of the correlation properties of biphase code CW signal on the range axis. The Doppler resolution property of the signal is the same as coherent pulse train on the frequency axis, but these are higher sidelobe levels. It could be improved apparently by using weighting functions to compensate echo signal; however, sidelobe levels of range ambiguity function would be worse.

A new measurement method is proposed. First, a precise range measurement value would be achieved through demodulating MPPSK pulse echo signals. Second, using the weighted algorithm, a precise velocity measurement value would be obtained by processing the weighted data. After the integration of the two data, the single-frequency MPPSK integrated system has the capability of wide range and high precise velocity and ranging measurement.

## 6. Conclusions

Combining the merits of biphase code CW signal base on *m* sequence and coherent pulse train, HRF biphase code MPPSK pulse train base on *m* sequence is proposed in this paper. The proposed signal can be summarized as the following characteristics.

1. The proposed signal succeeds many of the advantages of biphase code CW signal. Also, a solution is offered to solve the problem of transmit-receive community antenna.
2. It has high resolution both in range and in velocity measurement. In particular when compared with coherent pulse train, the selection of different software-hardware combination schemes makes it possible to improve measurement accuracy and range, and different accuracy for different signal segments can also be achieved. The simulation experiment results show that the proposed signal can enhance the ability of resisting noise interference.
3. The sampling rates of biphase code CW radar system and single-frequency MPPSK integrated system are different. The sampling rate of the former is bit rate while the latter is the equivalent width of single MPPSK pulse.
4. In addition to the high ranging and velocity resolution, the system is stable and offers strong anti-interference capability and reliable target detection ability in long distance.

## Figures and Tables

**Table 1:** Simulation parameter.

Parameter value

Carrier frequency: *f*_1_ = 10 GHz

Modulation parameter: *K* = 1,3, 5,10, *N* = 100, *M* = 4
*L* = 5 × 10^4^

Receiver distance: 1080 m

**Table 2:** Simulation parameters.

Parameter value
Carrier frequency: *f*_1_ = 10 GHz
Modulation parameters: *N* = 30, *M* = 4
